# DSP-Assisted Nonlinear Impairments Tolerant 100 Gbps Optical Backhaul Network for Long-Haul Transmission

**DOI:** 10.3390/e22091062

**Published:** 2020-09-22

**Authors:** Muhammad Irfan, Farman Ali, Fazal Muhammad, Usman Habib, Abdullah S. Alwadie, Adam Glowacz, Ziaul Haq Abbas, Eliasz Kańtoch

**Affiliations:** 1 Electrical Engineering Department, College of Engineering, Najran University Saudia Arabia, Najran 61441, Saudia Arabia; asalwadie@nu.edu.sa; 2 Department of Electrical Engineering, Qurtuba University of Science and IT, D. I. Khan 29050, Pakistan; drfarmanali.optics@qurtuba.edu.pk; 3 Department of Electrical Engineering, City University of Science and Information Technology, Peshawar 25000, Pakistan; 4 School of Electrical Engineering, Korea Advanced Institute of Science and Technology, Daejeon 34141, Korea; engrusmanhabib@gmail.com; 5 Department of Automatic, Control and Robotics, AGH University of Science and Technology, 30-059 Krakow, Poland; 6 Faculty of Electrical Engineering, GIK Institute of Engineering Sciences and Technology, Topi 23640, Pakistan; ziaul.h.abbas@giki.edu.pk; 7 Department of Biocybernetics and Biomedical Engineering, Faculty of Electrical Engineering, Automatics, Computer Science and Biomedical Engineering, AGH University of Science and Technology, Al. A. Mickiewicza 30, 30-059 Kraków, Poland; kantoch@agh.edu.pl

**Keywords:** optical fiber communication, wavelength division multiplexing (WDM), nonlinear effective area, digital signal processing (DSP)

## Abstract

High capacity long haul communication and cost-effective solutions for low loss transmission are the major advantages of optical fibers, which makes them a promising solution to be used for backhaul network transportation. A distortion-tolerant 100 Gbps framework that consists of long haul and high capacity transport based wavelength division multiplexed (WDM) system is investigated in this paper, with an analysis on different design parameters to mitigate the amplified spontaneous emission (ASE) noise and nonlinear effects due to the fiber transmission. The performance degradation in the presence of non-linear effects is evaluated and a digital signal processing (DSP) assisted receiver is proposed in order to achieve bit error rate (BER) of 1.56 × $10^{-6}$ and quality factor (Q-factor) of 5, using 25 and 50 GHz channel spacing with 90 μm${}^{2}$ effective area of the optical fiber. Analytical calculations of the proposed WDM system are presented and the simulation results verify the effectiveness of the proposed approach in order to mitigate non-linear effects for up to 300 km length of optical fiber transmission.

## 1. Introduction

An important aspect of a long haul optical fiber system is to transmit ultra-high data rate throughput over long distances and minimize the number of signal repeaters or regenerators, which usually requires optical amplification after regular intervals of transmission. The revolution in long haul transmissions and adaptation of wavelength division multiplexing (WDM) is certainly due to the erbium doped fiber amplifier (EDFA) [1]. The associated amplified spontaneous emission (ASE) noise from the EDFA and transmission fiber nonlinearities are the major limitations for such systems to achieve high data rates in optical communication system over long distances [2]. Another major source for nonlinearities is the use of high input optical power level which is required to minimize the number of repeaters in a long haul link [3]. Because of the components that have nonlinear transfer function, the aggregated distortion to the signal becomes manifold and, hence, causes severe performance degradation [4,5]. Cross phase modulation (XPM) [6], four wave mixing (FWM) [7], and self phase modulation (SPM) [8] are the key nonlinear impairments in optical fiber communication system, which needs to be mitigated for reliable and error-free transmission. As fiber transmission impairments are generally associated with the analog transmission over the fiber [9], the digital modulation also undergoes performance degradation due to the accumulated non-linear effects. Recent research work in this domain include the use of digital Nonlinearity Compensation (NLC) in a fully loaded WDM multiplexed transmission system [10], the shaping algorithm for mitigating the effect of nonlinear phase noise in fiber optic transmission [11], the single step nonlinear impairment based on the Gaussian mixture model [12], and a complex equalizer for a 40 Gbps system to achieve transmission over 240 km optical fiber using a single channel [13]. However, these mentioned models have complex build up and cost restrictions. To overcome these limitations, we propose a cost effective and simple network structure for long haul high data rate system. The proposed architecture is tested for higher data rates, i.e., beyond 100 Gbps. Satisfactory results are achieved for high data rates, e.g., 1.56 × $10^{-6}$ bit error rate (BER) while using effective fiber area of 90 μm${}^{2}$ by keeping 25 GHz frequency spacing among WDM channels, which is an acceptable range for the design of future backhaul networks. The aggregated effect of ASE due to the multiple use of EDFA in each transmission span [14] and filtration effects due to the use of Fiber Brag Grating (FBG) at the WDM demultiplexer end is also considered to realize a practical long haul transmission system [15].

### Major Contribution

The main purpose of this paper is to investigate the nonlinearities due to the optical fiber, which limits the transmission of ultra-high data rate data through long distances, and to present a low-complexity, low-cost solution through analytical modeling and simulation analysis. The major contribution of this paper are listed, as follows.

1. An analytical model is presented with a simple DSP receiver to compensate nonlinearities for long distance optical networks and support 8, 16, and 32 WDM channels in the presence of nonlinearities. The results validate that 32 channels supporting 100 Gbps can be easily supported up to 300 km of optical fiber transmission.
2. The performance affecting parameters such as nonlinear effective area, nonlinear refractive index, launch power, and length of fiber are used for a range of values in simulation analysis to analyze the effectiveness of the proposed technique.
3. A mathematical model for the nonlinear effects and their compensation through the proposed DSP technique is presented as the main contribution of this work. The proposed transmission model is compared for a range of nonlinearities, and the results are compared with an uncompensated case to quantify the improvement in the performance.
4. The BER and Optical Signal-to-Noise Ratio (OSNR) parameters are used as performance metrics to demonstrate the results of the proposed model. The achieved BER and OSNR are found to be $1.56\times 10^{-6}$ and 25, respectively.

The remaining part of this paper is divided into following sections. Section 2 consists of the description of the proposed model with a block diagram. An analytical model explanation in terms of nonlinear parameters is explained in Section 3. Discussion of simulation results are presented in Section 4. Section 5 concludes the presented work.

## 2. Proposed Network Architecture

**Figure 1:**
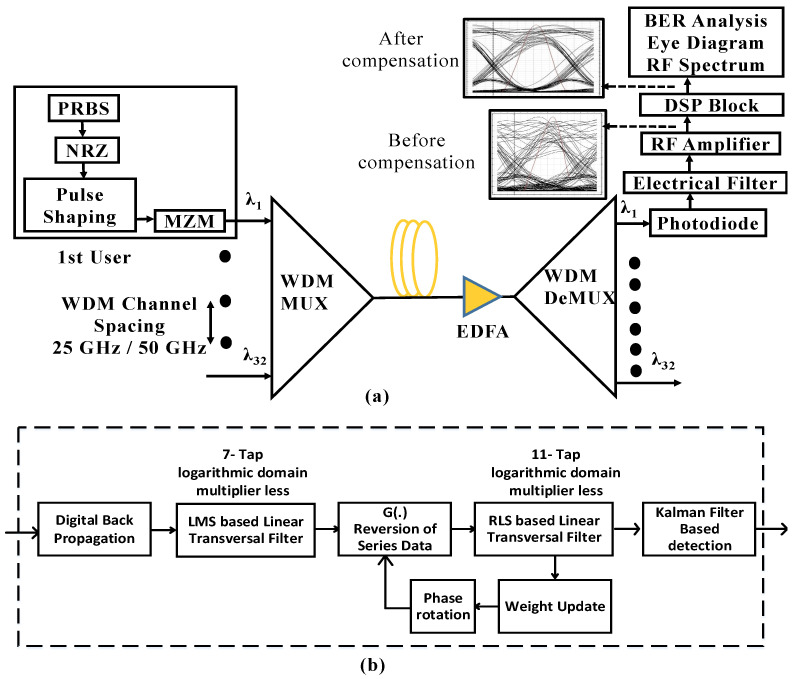
(**a**) Considered setup for the optical fiber communication system for beyond 100 Gbps data rates; (**b**) detailed block diagram of the proposed DSP receiver.

The aim of this work is to compensate the effect of the nonlinearities while using a cost effective model. This section includes the proposed architecture of the long haul high capacity optical network, for which the considered downlink setup is shown in Figure 1.

Figure 1a shows a WDM transmitter consisting of an array of independent free running lasers separated by a fixed channel spacing (25 or 50 GHz). External modulation has been used in this work to achieve high extinction ratio with minimum nonlinear effects from the transmitter side. The use of an electroabsorption modulated laser (EML) causes nonlinear effects due to the limited linear range of operation and transient chirp of the laser. On the other hand, directly modulated laser (DML) based systems experience further degradation in performance due to the intrinsic adiabatic chirp. To compensate the chromatic dispersion effects, duo-binary modulation has been used in this work, which is a three level signal in electrical form (instead of two as used in many other modulation formats and waveform). The optical form of the duo-binary modulation is achieved by over-driving a Mach-Zehnder modulator (MZM) after producing the driver signal by passing the output of the pseudo random bit sequence (PRBS) module through a low pass filter. The low pass filter acts as a duo-binary encoder and it produces the same effect in frequency domain as a one-bit delayed line in electrical domain to produce a set of correlated signal levels for the duo-binary modulation. The duo-binary modulation occupies less spectrum and therefore is more tolerant the fiber dispersion effects, which is very useful for long haul transmission. A modulator driver is used with the MZM to control the bias voltage and ensure a full swing of 2V${}_{pi}$ swing is available for the modulation. A WDM Mux is installed to transmit optical signals over single mode fiber by combining different wavelengths with an insertion loss of 3 dB. The transmission fiber consist of 0.2 dB/km constant loss and, to compensate the power loss, an EDFA amplifier is used in the system after each 100 km of transmission loop. At the receiver side, after de-multiplexing, the optical signals are converted into electrical signals by employing the photo detectors for direct detection that converts the duo-binary modulated data to a simple NRZ demodulation. The BER analyzer is used to quantify the performance of the received signals. In the Figure 1, a sub-figure is also presented to depict how the transmitted various pulses spread inside single mode fiber. The performance is evaluated by keeping $5\times 10^{9}$ symbol/s symbol rate for eight and 16 number of channels, thus providing aggregate data rate of 40 Gbps and 80 Gbps. For 32 number of channels, symbol rate has to be dropped to $3.2\times 10^{9}$ symbol/s to satisfy the BER limit of $3.8\times 10^{-3}$, which provides over 100 Gbps data rate. The simulations are performed in a high performance software Optisystem, which is a well-known tool for optical communication system analysis. Optisystem provides a large set of optical and RF components library to enable users for modeling of a complete downlink or uplink system. The range of signal visualizers and analysis tools makes it a useful resource to investigate the practical system designs.

Figure 1b shows the details of the proposed DSP receiver. To improve the performance of the system in the presence of second-order non-linearities, a cascade operation of LMS based linear transversal filter (seven-tap) and RLS filter (11-tap) is used. This function combines the advantages of the two approaches by improving the convergence time, even in the presence of non-stationary impairments in the channel. The number of taps are optimized for the system presented in Figure 1a for the minimum least square error (LSE) and fastest convergence time. In order to avoid the use of training sequence and complexity of the overall transmission, blind adaptive equalization is employed using constant modulus algorithm (CMA). As nonlinearities present in the system are multiplicative in nature for long haul transmission distances, a Kalman filter approach is used instead of matched filter for the detection of received bits. It uses a likelihood thresholding ratio (LTR) approach for the detection of bit levels, and updates threshold after each iteration.

Table 1 summarizes the list of different parameters and their values used in the considered model. The three sub-sections show the values used at the transmitter side, optical propagation channel, and the receiver. For the simulation work, most of the parameters are kept constant to ensure a fair comparison between the conventional and the proposed system, while some important set of values is used as a range for the factors affecting the performance of the system during the transmission, such as nonlinear refractive index, nonlinear effective area, WDM spacing, linear and nonlinear dispersion, and dispersion slope. The optimized performance is achieved 1553 nm reference wavelength, $2.6\times 10^{-17}$ refractive index, and 90 μm${}^{2}$ nonlinear effective area of the fiber for long haul optical communication system. For a realistic approach towards the practical system design and analysis, the set of values shown in Table 1 have been taken from real time field trials and experimental work in this domain. The optical and RF components are modeled with commercially available devices [9,15] and nonlinearities are included using the measured set of values [16,17].

## 3. Analytical Model

In Section 2, the proposed model is described based on a block diagram and system model used for the simulation work. This section discusses the basic theoretical model of the proposed work for 100 Gbps (and above) optical fiber communication network system with including nonlinear issues. The effect of nonlinearities due to the fiber transmission and their mitigation using the proposed receiver are explained, as follows: The output of the laser source is given by [18,19,20]

(1) $$F_{o}=F_{i}^{j\omega ct}$$

(2) $$E_{o}=E_{i}/2\left[e^{\left(i\pi v_{1}\left(t\right)\right)/v_{\pi }}+\gamma e^{\left(\left(i\pi v_{2}\left(t\right)+v_{bias}\right)/v_{\pi })\right)}\right]$$

The optical fields are modulated by modulator using the pulses that are induced by the medium, explained below as [16]

The $V_{1}$ and $V_{2}$ are given as

(3) $$V_{1}=-V_{2}=\left(P\left(t\right)\right)/2$$

The variables used in the above equations are input optical field denoted by $E_{i}$, $V_{\pi }$ shows the magnitude of the modulator, bias of the modulator circuit explored by $V_{bias}$, $V_{1}$ and $V_{2}$ explain the electrical pulses voltage, $F_{i}^{j\omega ct}$ denotes the laser output and coefficient of the refractive index denoted by γ. Mathematical explanation of the γ is presented by Equation (4) [21]

(4) $$\gamma =\left(\pi N_{2}\right)/\left(\lambda A_{e}C\right)$$

Here $N_{2}$, $A_{e}$, λ, and *C* denote non-linear regime of the compensating fiber, effective area of the transmitting fiber, wavelength, and weighing constant, respectively. The generalize model of the Schrodinger equation can be written as [17,22]

(5) $$\begin{array}{c} \partial U/\partial Z+\alpha /2U-\sum_{\left(i\geq 2\right)}n^{(i+1)/i!}\beta_{i}\left(\partial^{i}U\right)/\left(nT_{i}\right)=\\ n\gamma (1+n\tau_{i}\partial U/\partial T) \end{array}$$

where *U* is the complex magnitude of the electric field, *T* define the retarded time, α is the fiber impairments, and $\beta_{i}$ is the linear and nonlinear dispersive term with function of *z*. The $\beta_{2}$ presents linear term, while $\beta_{3}$ denotes nonlinear dispersion. The induced polarization by electric dipoles is given by [23,24]

(6) $$p=\varepsilon_{0}\left(x^{n}E_{n}\right)$$

The parameter $\varepsilon_{0}$ is permittivity of free space and equal to 8.85×10${}^{-12}$F/m, $x^{n}$ explains order of susceptibility and n is equal to 1, 2, 3, ... *N*. The final element $E_{n}$ denotes the electric field. Initial term of the nonlinearity starts from $x^{3}$ and it is called as third order susceptibility, which concludes that both nonlinearity and order of susceptibility can be mitigated by a simple estimation of the third order nonlinear transfer function. The relation of the number of channel, *N*, with these parameter is expressed [21,22], as follows.

(7) $$N=n\left(\omega \right)+n_{2}{\left|E\right|}^{2},$$

where ${\left|E\right|}^{2}$ is called nonlinearity coefficient, which is material dependent and it is related to third order susceptibility.

(8) $$N=n\left(\omega \right)+\frac{n_{2}P}{A_{eff}},$$

where n(ω), *P*, and $A_{eff}$ describe linear refractive index, launch power (The terms launch power and input power are used interchangeably in this paper.), and nonlinear effective area, respectively. In long haul optical networks, a complex modulated signal contains information in its phase as well as amplitude [25,26,27,28] can be represented as

(9) $$E_{s}\left(t\right)=\zeta_{s}\left(t\right)\varrho^{j(\psi t+\forall t)},$$

where $E_{s}\left(t\right)$ is a complex modulated signal, $\zeta_{s}\left(t\right)$ represents amplitude component of the signal, the carrier frequency of the signal is denoted by ψt, and parameter ∀t means time dependent phase. The power of the propagated optical signal is given as

(10) $$p_{i}\left(t\right)=p_{x}+p\left(t\right)sin\left[\Delta \theta t+\Delta \beta \right],$$

where $p_{i}\left(t\right)$ and p(t) are define, as follows

(11) $$p\left(t\right)=2\zeta_{(}t)\zeta_{s}\left(t\right),$$

(12) $$p_{i}\left(t\right)=\zeta^{2}\left(t\right)+\zeta_{s}^{2}\left(t\right).$$

where Δθ and Δβ explain frequency and phase of the signal respectively. The attenuation, α, losses inside the fiber [18] is given as

(13) $$\alpha \left(l\right)=\frac{10}{l}\left(\frac{\varepsilon_{out}}{\varepsilon_{in}}\right),$$

where $\varepsilon_{out}$ and $\varepsilon_{in}$ present output and input power values of optical fiber and *l* is the fiber length. Similarly, the dispersion effects are given as

(14) $$\Omega =\frac{2\pi c}{\chi^{2}}\vartheta_{2},$$

(15) $$\Psi =\frac{4\pi c}{\chi^{3}}\left(\vartheta_{2}+\frac{\pi c}{\chi }\vartheta_{3}\right).$$

Here, $\vartheta_{2}$ and $\vartheta_{3}$ mean second order and third order dispersion coefficients respectively, Ω is group velocity dispersion, Ψ denotes dispersion slope, *c* represents the speed of light and wavelength of the pulse [29,30] is represented by χ.

To deduce the computational requirements, implementing multi-span, the simulation model is calculated using BER, which is working based on following equation

(16) $$\mathrm{BER}=Q\sqrt{2\varphi }.$$

Here φ defines OSNR per bit and the function of *q* is calculated as

(17) $$Q\left(n\right)=\frac{1}{2\pi }\int_{1}^{\infty }exp\left(-n^{2}/2\right)dn.$$

where *n* denotes the number of bits received in error.

## 4. Simulation Results

In the above sections, the proposed model and its analytical approach are presented in order to show that nonlinear effects can be mitigated using a simple DSP design of the receiver. In this section, based on the theoretical model, the simulations results are investigated in order to explore the performance of the proposed work.

**Figure 2:**
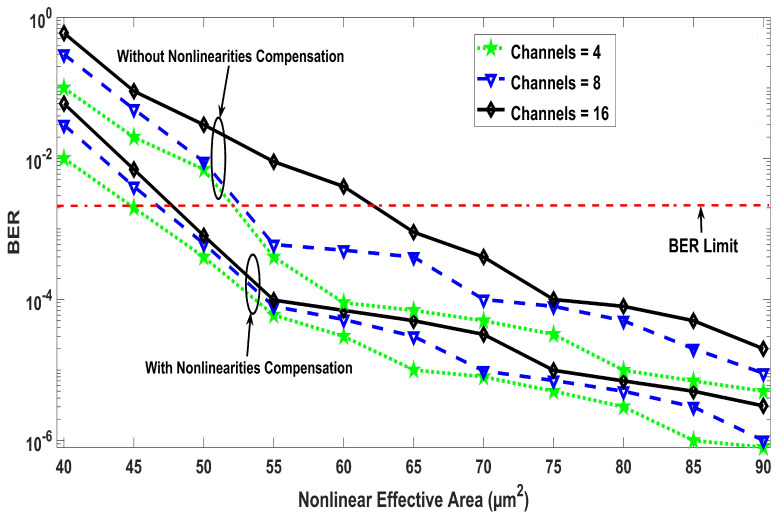
Nonlinear effective area against bit error rate (BER) for different channels and symbol rates.

**Figure 3:**
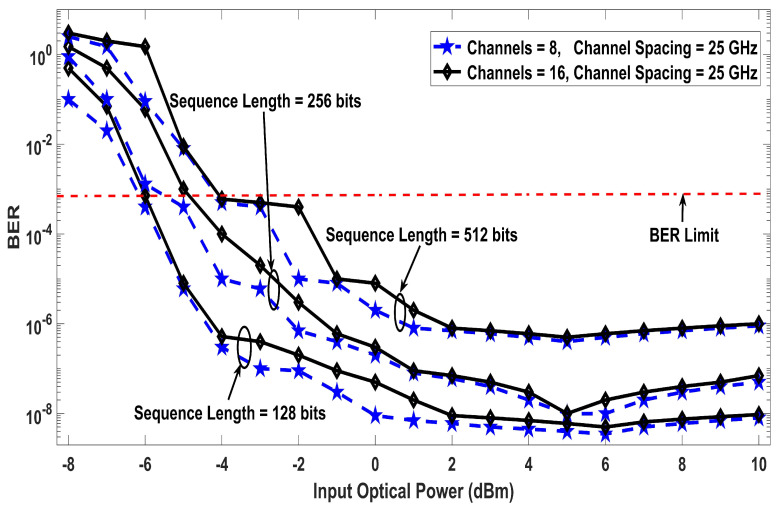
Effect of nonlinearities for different sequence lengths versus input optical power against BER, using eight and 16 channels (exclusively) with 25 GHz channel spacing.

**Figure 4:**
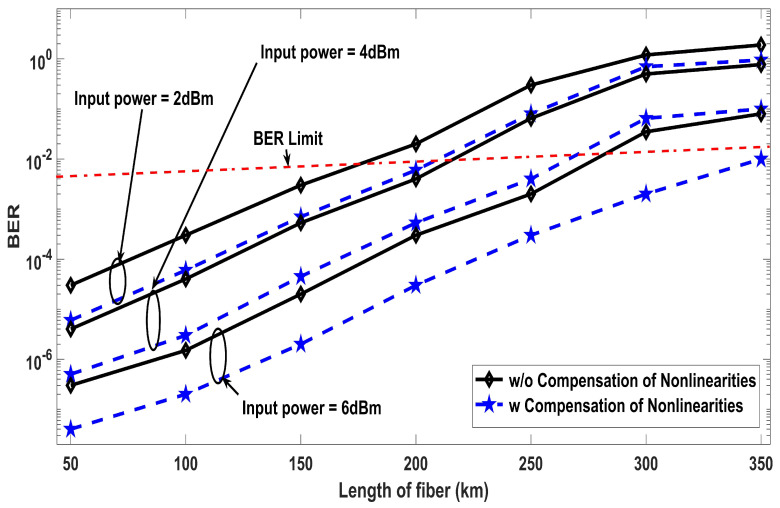
BER performance comparison of with and without nonlinearities compensation at 2, 4, and 6 dBm launch power in terms of different transmitted fiber lengths.

The results shown in Figure 2 are based on different values of nonlinear effective area against BER for four, eight, and 16 number of channels and $5\times 10^{9}$ symbol/s symbol rate. Moreover, the results clearly depicts that the proposed work has optimum performance at 90 μm${}^{2}$ nonlinear effective area for all considered cases but the proposed receiver design effectively mitigates the nonlinear effects to a considerable extend. For instance, the same BER is achieved with 16 number of channels with the nonlinearities compensation, as with four channels without applying the compensation. Thus, the number of channels can be increased by four times with the proposed solution in cases where the system’s performance is affected due to the nonlinearities. When considering the FEC limit of BER ($3.8\times 10^{-3}$), only 48 μm${}^{2}$ is required for all cases after the application of the nonlinearities compensation, which otherwise requires 53 μm${}^{2}$ for a four channel system and 63 μm${}^{2}$ for 16 channel system. The simulation results of the proposed model investigated in terms of input power against BER are mentioned in Figure 3 by putting several sequence length of bits. The transmission distance of 300 km has been considered here and it can be seen that the system performance improves with the increase in power up to a point where the nonlinearities are compensated by the DSP receiver. The optimum results are achieved at input power levels of 6 dBm, after which the BER increases even with the applied compensation, because the interaction of such high power level with the nonlinear optical fiber medium generates additional third order distortions. The transmit power of 6 dBm into the fiber is essential to achieve longer transmission distances. Figure 3 also shows that increasing the length of sequence results in broadening of the nonlinearities. The analysis for different sequence length shows that the sequence length dependent autocorrelation function amplitude doe not affect the effectiveness of the proposed compensation technique and low BER can be achieve with any sequence length without the loss of generality. Figure 4 demonstrates an in-depth details of the results with a range of fiber lengths against BER at 2, 4, and 6 dBm input launch power levels. With the proposed compensation and 6dBm launch power, the transmission distance up to 300 km satisfies the BER limit of $3.8\times 10^{-3}$, which clarifies that the proposed system can be used for cumulative nonlinear effects due to long haul transmissions.

**Figure 5:**
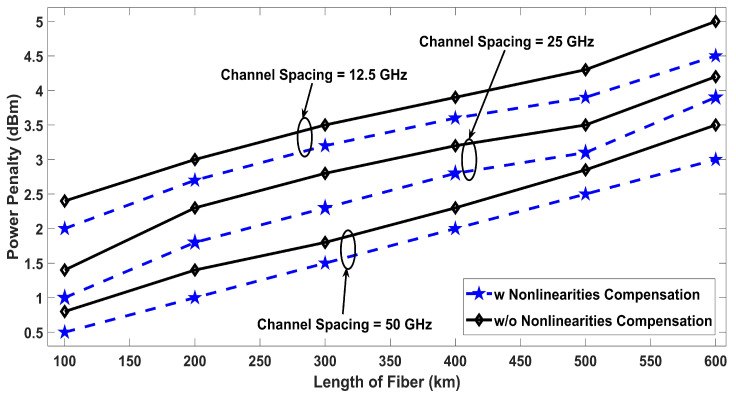
Power penalty due to nonlinearities at different channel spacings.

**Figure 6:**
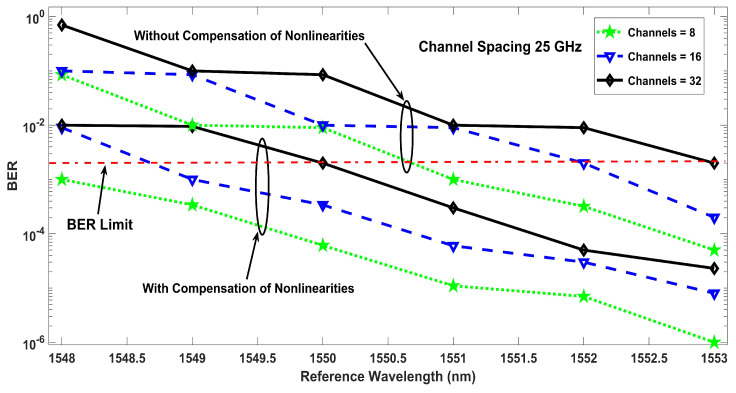
Reference wavelength against BER at different number of channels.

**Figure 7:**
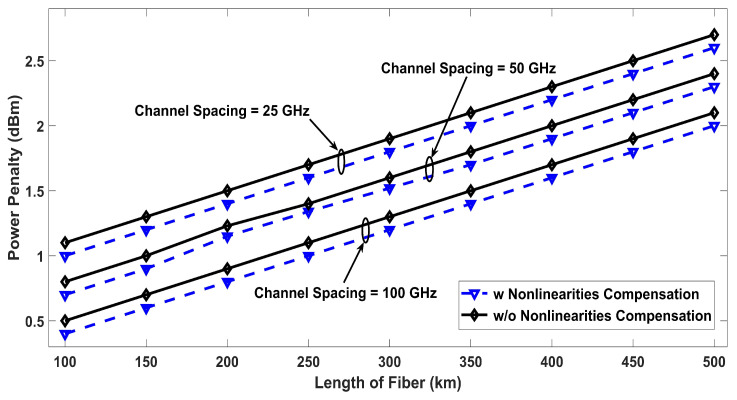
Power penalty at various transmitted fiber lengths using −3, −2, and −1 ps${}^{3}$/km nonlinear dispersion.

**Figure 8:**
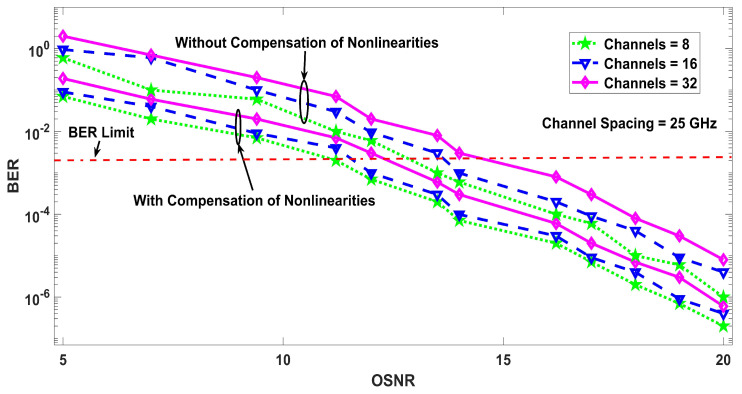
BER performance for different values of available optical signal-to-noise ratio (OSNR).

Similarly, Figure 5 defines the performance of the proposed model with nonlinearities compensation as an untreated transmission with nonlinearities. In addition to the simulation results of the proposed model showing BER performance, Figure 5 describes the amount of power penalty due to nonlinearites at 12.5, 25, and 50 GHz channels spacing, which declares that the performance of the compensated system is better, but a penalty has to be paid for having low channel spacing, which is often required to accommodate large number of channels within a specific frequency span. Figure 6 shows the effect of reference wavelength, where the BER results are shown as a function of the reference wavelength for eight, 16, and 32 channels, where it can be seen that multiple nonlinearities depend on the reference wavelength and the proposed model has greater tolerance against nonlinearities with a general optical fiber transmission system. The symbol rate of 5 GSym/s has been used for eight and 16 channels which provides a data rate of 40 Gb/s and 80 Gb/s, respectively. For 32 number of channels, the symbol rate has to be dropped to 3.2 GSym/s to ensure that the BER performance is under the limit. Figure 7 discusses the power penalties due to nonlinearities at −3, −2, and −1 ps${}^{3}$/km nonlinear dispersion. The system performance in terms of BER for various values of available OSNR is presented in Figure 8, in the presence of nonlinearities. The results show that lower values of BER can be achieved at low OSNR values using the proposed compensation technique.

**Figure 9:**
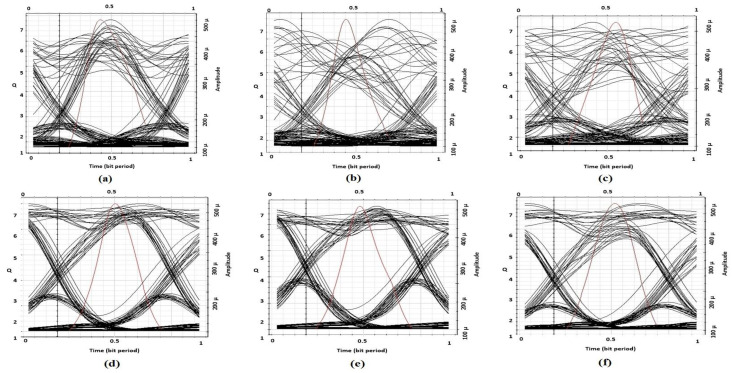
BER visualizer diagrams; (**a**): Without nonlinearities compensation at 8 channels, (**b**): Without nonlinearities compensation at 16 channels, (**c**): Without nonlinearities compensation at 32 channels, (**d**): With nonlinearities compensation at 8 channels, (**e**): With nonlinearities compensation at 16 channels, (**f**): With nonlinearities compensation at 32 channels.

The performance behavior of the proposed set up is explored based on eye diagram analyzer and visual inspection of the RF analyzer. Figure 9a–c represent the eye diagram at the receiver end, when eight, 16, and 32 channels are used for the considered WDM system, respectively, where no compensation technique is applied to mitigate nonlinearities. Thus, the look of the eye diagrams are worse which defines that error ratio is high in the received signals as the number of channels are increased. While, Figure 9d–f eye diagrams denote the proposed model outcomes for 8, 16, and 32 channels respectively, which show the improvement in performance with the presented DSP technique. The quality factor (Q-factor) is measured from the eye diagrams to be five using the quantitative measurement methods provided in [31]. The Q-factor is estimated by taking the ratio between sum of eye opening (decision point to the edge) to the sum of RMS noise at the edge.

**Figure 10:**
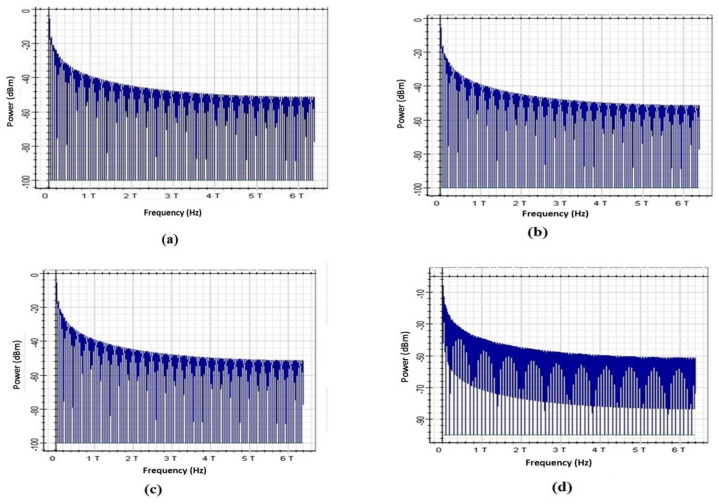
RF visualizer diagrams; (**a**): Without nonlinearities compensation at 16 channels, (**b**): Without nonlinearities compensation at 32 channels, (**c**): With nonlinearities compensation at 16 channels, (**d**): With nonlinearities compensation at 32 channels.

The RF spectrum visualizer is used for calculating and displaying electrical signals in the RF frequency domain. It can display the signal intensity and power spectral density. Figure 10a–d depict the improvement with the involvement of DSP module for 16 and 32 number of channels. Figure 10a,b diagrams present the received spectrum after the long haul transmission of the considered high capacity optical network without the compensation of nonlinearities, while Figure 10c,d explain the system performance after the application of the proposed DSP technique to compensate nonlinearities. The blue trace represents the signal and grey trace shows the nonlinear noise. The reduction in nonlinearities can be clearly observed by comparing Figure 10a with Figure 10c that the amplitude of nonlinear noise is reduced by up to 15 dB. From the detailed discussion of the simulation results in this section, it is concluded that, the with the help of low cost and simple proposed model, the results show that high data rates beyond 100 Gbps can be easily transmitted up to 300 km range of length with low BER.

As discussed earlier, the simulation analysis has been performed by modeling the optical and RF components with the realistic values from the referenced work on field trials and experimental work in this domain. The proposed solution for the compensation of nonlinearities does not require additional hardware and it can easily be implemented in the real-time systems using DSP assisted receiver. The projections made by the presented simulation analysis to transport of 100 Gb/s data rate over 300 km of transmission distance can be really useful for design engineers to extend the current optical backhaul lengths and upgrade the data rate capacity.

## 5. Conclusions

Long haul and high capacity communication for the backhaul network are fundamental requirements of the modern communication systems, facilitating users with low cost, reliable, and highly efficient transmission. This work investigates a 100 Gbps data rate WDM system for optical fiber long haul transmission with the help of advance DSP approaches in order to minimize the complexity and cost of the receiver and tolerate the accumulated nonlinearities. The proposed model is explored on the basis of the presented analytical model to compensate the nonlinear effects for different number of WDM channels, and frequency spacing among them, for transmission distances up to 300 km. Moreover, the proposed model is compared with a general uncompensated transmission to show the effectiveness of the proposed approach. The power penalty is also discussed in this paper, due to the nonlinear effects and the improvement with the DSP receiver is quantified for a range of parameters, such as nonlinear effective area, input power, and reference wavelength. For the WDM transmission, four, eight, 16, and 32 number of channels are considered with various channel spacings of 12.5, 25, and 50 GHz for high launch power levels and considering the ASE noise from the EDFA, and the results show that the proposed setup successfully mitigates the consequent impairments and provides low BER and power penalty. 

## Figures and Tables

**Table 1:** Parameters used at the transmitter end.

Subsystem	Component	Parameter	Value
Transmitter	CW laser	frequency	193.1 THz with 25 and 50 GHz channel spacing
	Linewidth	frequency	1 MHz
	Total Power	Launch power	6 dBm
	MZM	Insertion Loss	5 dB
	MZM	Half-wave RF voltage (Vπ)	5 V
	Channel	Quantity	32
	Pulse generator	NRZ	aggregate 100 Gbps
	WDM Mux/DeMux	Insertion loss	3 dB
	25/50 GHz spacing filter	Optical bandpass bandwidth	5 GHz
Optical link	Fiber	Transmission Length	300 km
		Attenuation	0.2 dB/km
		Reference wavelength	1553 nm
		$\beta_{2}$	−18 ps${}^{2}$/km
		$\beta_{3}$	−3 ps${}^{3}$/km
		Nonlinear refractive index	2.6×10${}^{-17}$ m${}^{2}$/W
	Amplifier	EDFA Gain	30 dB
	Impairments	Dispersion	17 ps/nm
	Filter	Optical Filter order	8
Receiver	Photo-detector	Responsivity	1 A/W
	Low pass filter	Cutoff frequency	0.75× bit rate Hz
